# In Vitro Fermentation of Beechwood Lignin–Carbohydrate Complexes Provides Evidence for Utilization by Gut Bacteria

**DOI:** 10.3390/nu15010220

**Published:** 2023-01-01

**Authors:** Xiaochen Ma, Shujun Liu, Hongliang Wang, Yulu Wang, Zhen Li, Tianyi Gu, Yulong Li, Fengjiao Xin, Boting Wen

**Affiliations:** 1 Laboratory of Biomanufacturing and Food Engineering, Institute of Food Science and Technology, Chinese Academy of Agricultural Sciences, Beijing 100193, China; 2 Center of Biomass Engineering, College of Agronomy and Biotechnology, China Agricultural University, Beijing 100193, China

**Keywords:** LCC, SCFAs, gut microbiota, metagenome, CAZymes

## Abstract

Lignin–carbohydrate complexes (LCCs) are emerging as a new and natural product with pharmacological and nutraceutical potential. It is uncertain, however, whether LCCs have a positive effect on the microbiota of the gut based on the current evidence. Here, the LCC extracted from beechwood (BW-LCC) was used as a substrate for in vitro fermentation. The lignin in BW-LCC consisted of guaiacyl (G) and syringyl (S) units, which are mainly linked by β-O-4 bonds. After 24 h of in vitro fermentation, the pH had evidently declined. The concentrations of acetic acid and propionic acid, the two main short-chain fatty acids (SCFAs), were significantly higher than in the control group (CK). In addition, BW-LCC altered the microbial diversity and composition of gut microbes, including a reduction in the relative abundance of Firmicutes and an increase in the relative abundance of Proteobacteria and Bacteroidetes. The relative abundance of *Escherichia coli-Shigella* and *Bacteroides* were the most variable at the genus level. The genes of carbohydrate-active enzymes (CAZymes) also changed significantly with the fermentation and were related to the changes in microbes. Notably, the auxiliary actives (AAs), especially AA1, AA2, and AA3_2, play important roles in lignin degradation and were significantly enriched and concentrated in Proteobacteria. From this study, we are able to provide new perspectives on how gut microbes utilize LCC.

## 1. Introduction

Human gut microbiota is host-specific, changes continuously during individual development, and is susceptible to external influences [1]. Among them, diet is a major determinant of microbiota structure by regulating its composition and functionality [2]. Dietary fibers, such as resistant starch, inulin, lignin, pectin, cellulose, hemicellulose, oligosaccharides, and so forth, have extensively existed in daily food and are abundant in cereals, vegetables, fruits, and other plant foods [3,4,5]. Fibers in dietary products are capable of resisting digestion and absorption by the small intestine, allowing them to reach the large intestine undigested. They could be metabolized by certain intestinal microflora in an anaerobic environment to produce substances such as short-chain fatty acids (SCFAs), thereby altering the composition of gut microbes to affect the body’s health [6,7,8]. The chemical characteristics of dietary fibers, which include polymerization degree, composition, and chemical bond form, have different effects on gut microbes [9,10,11]. 

Lignin–carbohydrate complex (LCC) is a type of dietary fiber and has been reported to have potential antitumor, antimicrobial, and antiparasitic biological activities [12,13,14]. The cross-linked structure of hemicellulose and lignin polyphenols makes it more resistant to utilization by intestinal microbiota. Hemicellulose is metabolized by intestinal flora to produce SCFAs and modifies gut microbiota composition [10,11,15]. In vitro studies revealed that lignin was an obstacle, as high-lignin content was more slowly digested by gut microbiota compared to low-lignin content [16]. Currently, in vitro fermentation of LCC from different plant sources has revealed that LCC could be utilized as a substrate to generate SCFAs and aromatic compounds and increase the growth rate of gut microbiota [17]. As of yet, it is not clear what effect LCC has on the composition of microbiota in the human gut.

Carbohydrate-active enzymes (CAZymes) can cleave complex high-polymerization carbohydrates into low-polymerization carbohydrates and more accessible components in the human gut [18]. The bioconversion of hemicellulose is associated with various classes of CAZymes, including glycoside hydrolases (GHs), glycosyltransferases (GTs), carbohydrate esterases (CEs), polysaccharide lyases (PLs), and Auxiliary Activities (AAs) [19]. AAs, a class recently added to the CAZy database (http://www.cazy.org, accessed on 22 November 2022), significantly contribute to the degradation of lignin [20]. The origin of AAs in the CAZy database is restricted to eukaryotes [20]. Thus, it is unknown how CAZyme species in gut microbes degrade LCC, especially the lignin components [21]. Additionally, the distribution of these CAZymes in gut microbes is still unknown. Gut microbes encode tens of thousands of CAZymes, and most are unidentified. This makes it a huge treasure trove of mining enzymes that can efficiently degrade lignocellulose [22].

The purpose of this study was to investigate the effect of LCC on the composition and abundance of gut microbiota and to investigate enriched CAZymes associated with LCC degradation. An analysis of the structure and composition of LCC derived from beechwood (BW-LCC) was performed using two-dimensional heteronuclear singular quantum correlation nuclear magnetic resonance (2D-HSQC-NMR). BW-LCC was fermented in vitro to investigate its influence on gut bacteria, as well as its physicochemical properties, including pH and SCFA yield. Phylum-level and genus-level changes in gut microbiota composition and abundance were examined with Illumina MiSeq high-throughput sequencing. The enrichment of CAZymes and the distribution of different families in gut bacteria was revealed through metagenomics. This paper provides a new perspective on the utilization of LCC by human intestinal microbiota from the two dimensions of microbiota and genes.

## 2. Materials and Methods

### 2.1. Extraction of BW-LCC

Beechwood samples were purchased from local farms. LCCs were separated from beech powder by a mechanocatalytical reaction system driven by fluid shear force, and the detailed procedure can be found in a previous study [23]. The procedure was performed as follows: 20 g of 40–60 mesh beech sawdust was soaked in 400 g of aqueous NaOH/urea solution and pre-cooled to −12 °C. The treated mixture was then fed into a high-rate agitator blender (Jess 100-Y) and fluid sheared at 49,000 r/min. The mixture was sheared for a total of 30 min, with 15 min breaks every 2–3 min to keep the temperature below 60 °C. 

### 2.2. Structural Characterization of BW-LCC

The extracted BW-LCC fractions were structurally characterized on an NMR spectrometer (Bruker spectrometer TYPE-AVIII 400 MHz, Bruker Co., Billerica, Massachusetts, USA). The LCC was dissolved in 0.75 mL of DMSO-d6 by sonication for 30 min. Subsequently, 2D HSQC NMR experiments were performed using Burker’s ’hsqcetgpsisp 2.2′ adiabatic pulse program with 4000 Hz and 20,000 Hz spectral widths in the ^1^H- and ^13^C-dimensions, respectively. Using a cryogenically cooled 3 mM HCN automated triple resonance probe and an Agilent DD2 600 MHz instrument, NMR spectra were recorded at 25 °C. The number of collected complex points was 2048 for the ^1^H-dimension, with a recycle delay of 1.5 s. ^1^H and ^13^C dimensions were processed using matched Gaussian apodization and squared cosine-bell apodization, respectively. The data matrix was Fourier transformed after 1024 points were filled in the ^13^C dimension. The central solvent peak was used as an internal reference.

The relative quantification of the lignin fraction based on 2D-HSQC-NMR was performed in Mestrenova11 using the integral (Int) for 2D HSQC spectra, where S = *0.5IntS_2,6_* and G = *IntG_2_*. Relative quantification of the linkage within BW-LCC was performed using the normalization method, the following equation was used to calculate the relative quantification of lignin linkage:

IX% = IX/(IA + IB + IC + ID) × 100% (1)

### 2.3. In Vitro Fermentation

The experimental method was performed as described by Chen et al. [9]. In vitro fermentation was carried out using 5 mL of YCFA medium with 1% substrate (*w*/*v*). The fermentation was carried out in syringed flasks with a total of ten parallel individual vials set up [24]. A mixture of N_2_ and CO filtered through a 0.2 μm PTFE membrane was used to create anaerobic conditions. Each vial was autoclaved at 121 °C for 20 min. After 24 h of incubation, the pH value of the supernatant was measured using a compact pH meter (B-212, Horiba, Kyoto, Japan). A total of 20 samples were divided into treatment and control groups, with 10 samples in each group. Samples were collected for the detection of SCFA production and bioinformatics analysis.

### 2.4. Determination of SCFAs

SCFAs were analyzed according to the methods of previous studies [9]. The method was as follows: 500 μL of fermentation broth was taken and the mixture was incubated with 100 μL of crotonic acid for 12 h at −20°C. The supernatant was centrifuged at 16,000× *g* for 3 min, and 100 μL of the supernatant was used for gas chromatography (GC) analysis. The specific method of GC was the same as previously described [9]. The final concentration is the yield of SCFA per unit weight of the substrate.

### 2.5. Determination of Microbial Diversity

The genomes of 20 sets of samples from in vitro fermentations were extracted using the E.Z.N.A. Soil DNA Kit (Omega Bio-Tek, Norcross, GA, USA). The V3-V4 highly variable region of the bacterial 16s rRNA gene was amplified by PCR with bacterial homologous primers 338F (5′-ACTCCTACGGGAGGCAGCAG-3′) and 806R (5′-GGACTACHVGGGTWTCTAAT-3′). Sequencing was performed using the Illumina Miseq sequencing platform of Shanghai Majorbio Bio-Pharm Technology Co. Based on a 97% uniformity threshold from the SILVA database, the resulting sequences were clustered into operational taxonomic units (OTUs) [25].

### 2.6. Metagenome Analysis

A total of 10 samples were selected for direct comparative metagenomic analysis, which was carried out at Majorbio Bio-Pharm Technology Ltd. (Shanghai, China) [26]. Annotation of carbohydrate-active enzymes (CAZymes) against the CAZy database was performed using hmmscan5 (http://www.cazy.org, accessed on 22 November 2022).

### 2.7. Data Statistics

Data on physicochemical properties were processed and analyzed using GraphPad Prism 8.4.2, and the results were expressed as mean ± SD. The results of the statistical analysis were analyzed using a one-way ANOVA and Kruskal–Wallis tests. Metagenome analysis and 16s rRNA sequencing were performed on the Bioinformatics Cloud platform, and all sequences were clustered according to the SILVA database’s 97% uniformity threshold and clustered into OTUs. When the *P* value was not greater than 0.05, the results were deemed statistically significant.

## 3. Results and Discussions 

### 3.1. Analysis of the Composition and Structure of Lignin in BW-LCC

#### 3.1.1. Structural Characterization of Lignin

**Figure 1:**
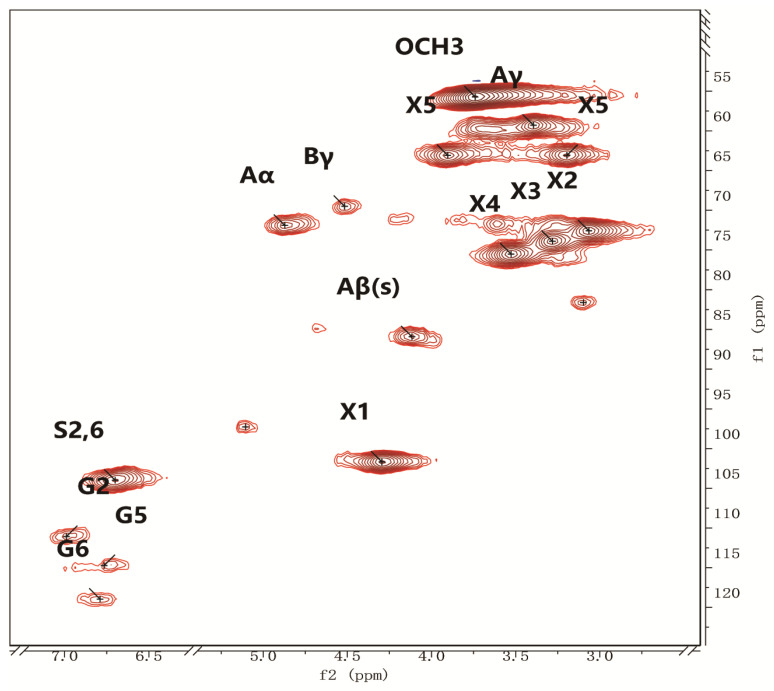
2D HSQC NMR spectrum of BW-LCC.

To determine the structural characteristics of BW-LCC, it was analyzed with 2D-HSQC-NMR, and the results are shown in Figure 1. In the NMR spectrum of BW-LCC, the lignin compound was divided into two main regions: the aromatic ring region (100–150 ppm/6–8 ppm) and the side chain region (50–90 ppm/2.5–6.0 ppm) [27]. In the aromatic region, the signal of the syringyl (S) unit was found at δC/δH =103.9/6.70 ppm, and the signals of the guaiacyl (G) units (G2, G5, G6) appeared at δC/δH = 110.8/6.97, 114.5/6.70, and 119.0/6.78 ppm, respectively. However, the p-hydroxyphenyl unit could not be detected, indicating that the sample was GS in type and compatible with the structural properties of broad-leaved plant lignin [28]. The full 2D-HSQC-NMR signal within the BW-LCC is shown in Tab.S1. Relative quantification of lignin unit content based on the results of the spectra was carried out according to Sette’s calculation method and the results are shown in Tab.S2 [29]. Among the lignin components, S units dominated (70.68% of the total), while G units accounted for just 29.32% of the lignin. Syringaldehyde contains good bioactive properties and plays an anti-oxidative, anti-cancer and anti-bacterial role [30,31]. 

#### 3.1.2. Quantification of Lignin Linkages

In the side chain of BW-LCC, it was noted that β-O-4 was the most usual linkage mode (Figure 1, Table S1), with additional β-β and other linkages also present. The α and γ potentials of β-O-4 are at δC/δH 71.8/4.86 and 59.9/3.35–3.80, respectively, while the potential of β-O-4 formed by an S-type unit is at δC/δH 85.8/4.12ppm. Besides the bonds mentioned above, other bonds were identified and summarized in Table S1. 

Comparing lignin–lignin linkages, the lignin–carbohydrate linkages provide insight into cross-linking between plant cell wall components. Different degrees of carbohydrate signals were found in the NMR side chain region of this lignin sample. Positions 1, 2, 3, and 4 of xylose are located at 101.5/4.25, 72.5/3.05, 73.7/3.25, and 75.3/3.56 ppm, respectively. The lignin portion of BW-LCC consists of an S-unit and a G-unit, with the lignin units linked to each other mainly by β-O-4 ether bonds and β-β, α-O-γ, and γ-O-α structures; the S-unit and G-unit are linked to the xylose components in the hemicellulose fraction by LCC bonds (Figure S1). 

### 3.2. pH and SCFAs Analysis

**Figure 2:**
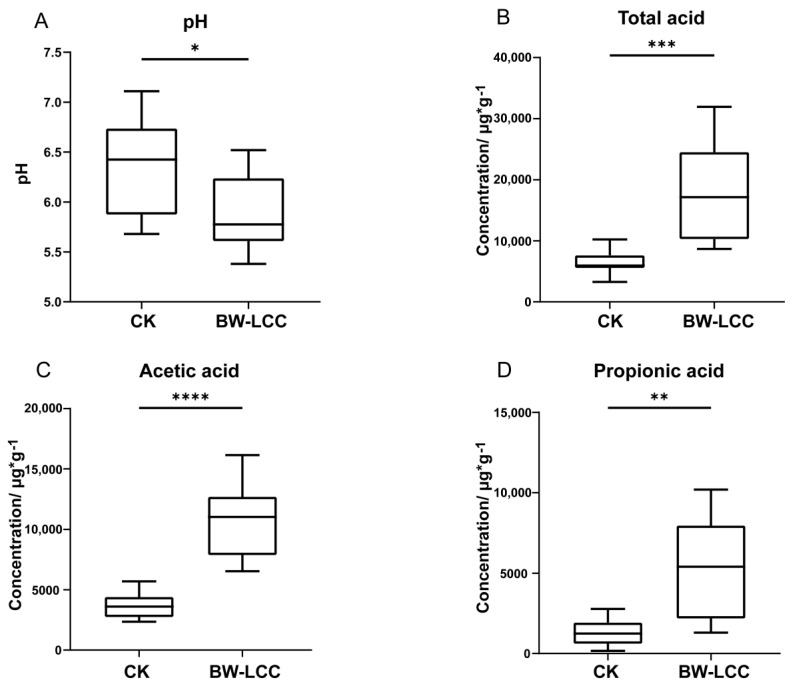
Changes of pH (**A**) and SCFA concentration compare with CK, including total acid (**B**), acetic acid (**C**) and propionic acid (**D**). *, *p* < 0.05, **, *p* < 0.01, ***, *p* < 0.001, ****, *p* < 0.0001.

The pH value is a key indicator of the growth of gut microbiota and the accumulation of metabolites during in vitro fermentation. Figure 2A shows the changes in pH values after 24 h of fermentation. Compared to the CK, the pH value was significantly decreased from 6.3 to 5.9 with BW-LCC as substrate. This suggests that BW-LCC can be utilized by intestinal flora and produces acids to lower the pH value in the fermentation broth. 

SCFAs are common products of dietary fiber degraded by intestinal microbiota. The accumulation of SCFAs can reduce intestinal pH value, which will bring further benefits to human health [32]. Meanwhile, the production of SCFAs is one of the most critical indicators for determining the utilization of dietary fiber by gut microbiota [6]. Here, the production of SCFAs was assessed by measuring the concentration of acetic acid, propionic acid, butyric acid, isobutyric acid, valeric acid, and isovaleric acid, and total acid represents the sum of all the above acids. However, the concentration differences between butyric acid, isobutyric acid, valeric acid, and isovaleric acid were too small to analyze. With BW-LCC as substrate, the concentrations of total acid, acetic acid, and propionic acid were significantly different from those of the control group (CK) (Figure 2B–D). After 24 h of fermentation, the yield of total acid (18,016 μg·g^−1^) in the BW-LCC group was significantly higher than that in the CK (** *p* < 0.01) (Figure 2B). The concentration of acetic acid reached 10,656 μg·g^−1^ after 24 h of fermentation. Acetic acid was the predominant SCFA in fermentation broth with BW-LCC as substrate and accounted for 68.73% of the total amount of acid (Figure 2C). It is reported that acetic acid is the richest SCFA in the gut and is therefore a crucial factor engaged in carbohydrates and lipid metabolism in the cell; acetic acid can also be absorbed by the liver for the synthesis of cholesterol [33]. As with acetic acid, the yield of propionic acid increased significantly (** *p* < 0.01) (Figure 2D) to a final concentration of 5267 μg·g^−1^. Propionic acid can improve insulin sensitivity and lowers hepatic and plasma concentrations of fatty acids, and this therefore might prove to be an elusive effector of obesity and type II diabetes [34]. Zhang et al. reported that LCC from different plants could be used as substrate for in vitro fermentation to increase the production of SCFAs [17]. This agrees with the results of the current study with BW-LCC as substrate. However, in vitro fermentation of BW-LCC did not promote butyrate production, and this could be due to structural differences between BW-LCC and other plant-derived LCCs. According to these results, BW-LCC is likely to contribute to the maintenance of gastrointestinal health. 

### 3.3. The Impact of BW-LCC on Gut Microbiota Communities

#### 3.3.1. Changes in Intestinal Microbial Diversity

**Figure 3:**
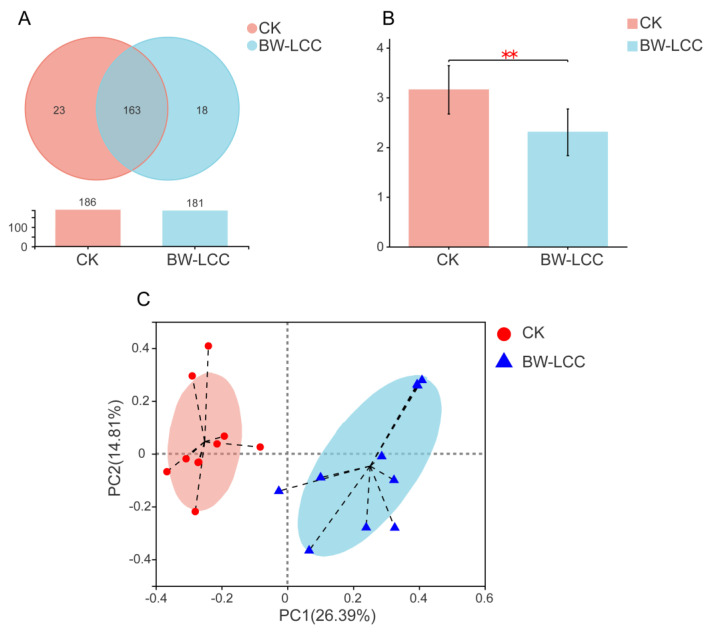
Diversity index of the intestinal microbial community after fermentation. (**A**) Venn diagram analysis at the OTU level. (**B**) The Shannon diversity index of microbial communities under each treatment at 24 h. Significance was determined between BW-LCC and CK using Wilcoxon rank-sum test; **, *p* < 0.01. (**C**) Principal Component Analysis (PCoA) based on diversity at the OTU level between the CK and BW-LCC groups.

The effect of BW-LCC on gut microbiota communities was revealed by 16s rRNA sequencing. After quality and chimaera checking, a total of 1,165,365 valid sequences were acquired from 20 samples, of which 10 samples were control (CK) and the rest were treatments (BW-LCC). The Shannon index is a common indicator that describes alpha diversity, and its value is positively associated with community diversity [35]. The Shannon curve tended to be flat but later obtained a stable pattern with an increased amount of data (Figure S2), thus indicating that the data obtained were sufficiently large to reveal the structural characteristics of the sample communities effectively. The similarities and differences in operational taxonomic units (OTUs) between the two groups were judged by Venn diagrams. A total of 204 operational taxonomic units (OTUs) were obtained from the CK and BW-LCC groups, including 163 common units, and the Venn diagram revealed the similarity between the two groups (Figure 3A). 

The Shannon index reflected a significant decrease in the alpha diversity of the BW-LCC group compared to the CK group after fermentation, which shows that BW-LCC may inhibit the growth of some intestinal bacteria and lead to the decline of microbial species. Based on a Principal Coordinate Analysis (PCoA) using the weighted UniFrac distance matrix, spatial separation and clustering of fermentative bacteria during fermentation with BW-LCC and CK treatments was demonstrated, with the two principal axes accounting for 42.2% of variation. This indicates that BW-LCC and CK separated from each other, and that the composition of the microbial community changed considerably.

#### 3.3.2. Effect of BW-LCC on Gut Microbiota Composition

**Figure 4:**
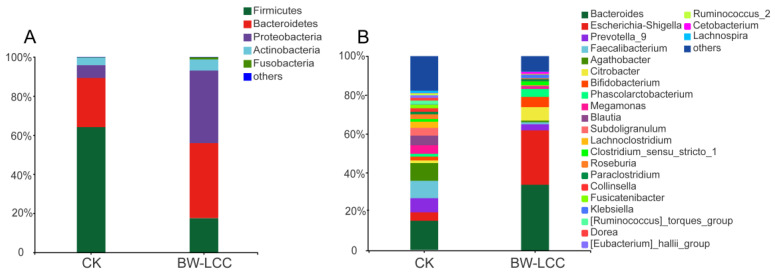
Comparison of microbial community composition between the BW-LCC and CK groups at the phylum (**A**) and genus (**B**) levels.

BW-LCC was compared to the CK group at the phylum and genus levels to assess its effect on different intestinal bacteria. The prominent bacteria in the CK group were identified as Proteobacteria, Firmicutes, Bacteroides, and Actinobacteria, which together accounted for about 99% of total gut bacteria. There was a significant increase in the relative abundance of Bacteroidetes and Proteobacteria in the BW-LCC group compared to the CK, which accounted for 38.37% and 37.10% of total gut bacteria, respectively (Figure 4A). In contrast, the relative abundance of Firmicutes was reduced to 17.56% after fermentation, as shown in Figure 4A. Proteobacteria were previously described as one of the most common bacterial species in the gut and are associated with a variety of diseases, thus causing inflammatory responses [36]. In a recent study, the Firmicutes/Bacteroidetes (F/B) ratio was identified as a critical biomarker of obesity, and a decrease in this ratio reduces the risk of obesity [37], suggesting that BW-LCC might prevent obesity by inhibiting Firmicute growth.

*Bacteroides* and *Escherichia-Shigella* showed the greatest increase in relative abundance at the genus level, reaching 33.89% and 28.00%, respectively, after fermentation (Figure 4B). The *Bacteroides* genome contains a large number of CAZymes genes, whose role is to help *Bacteroides* degrade complex carbohydrates in the gut, which are further metabolized by *Bacteroides* to produce SCFAs [38]. An increase in the percentage of Bacteroides may be responsible for the increased production of SCFAs. *Escherichia Shigella* is considered a pro-inflammatory bacterial species [39]. Combined with the results of previous studies, it is likely that fibers with a high degree of polymerization will increase the relative abundance of *Escherichia-Shigella* in gut microbes; however, this process may not be caused by butyric acid, and the specific reasons remain to be investigated [9]. The relative abundance of *Citrobacter* (6.90%), *Bifidobacterium* (5.29%), and *Phascolarctobacterium* (4.08%) also increased significantly. According to Niemi’s in vitro fermentation study, lignin-rich fractions did not inhibit the growth of *Lactobacilli* and *Bifidobacteria* [16]. In fact, there may be a more complex relationship between substrate LCCs containing lignin components and gut microbiota. 

### 3.4. Effect of BW-LCC on CAZymes

#### 3.4.1. BW-LCC Alters the Relative Abundance of CAZymes 

The NMR results showed that BW-LCC has a complex composition and structure, which requires synergistic degradation by multiple functional enzymes (Figure 1). These diverse CAZymes are not available in the human body itself, so these complex carbohydrates are only dependent on degradation by intestinal microbes [19,22]. To further understand the CAZymes enrichment after in vitro fermentation with BW-LCC, five samples from each of the BW-LCC and CK groups were taken for metagenomic analysis. The sequencing was optimized and screened to select the best splicing effect, and a total of 3,648,125 open reading frames (ORFs) were obtained by screening sequences larger than 100 bp. The obtained ORFs were translated into amino acid sequences, and the sequence distribution is shown in Figure S3.

**Figure 5:**
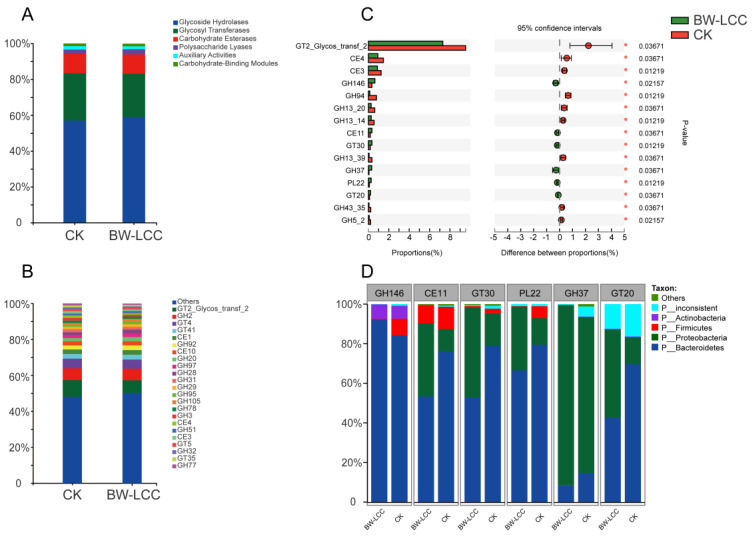
Comparison of CAZymes between the BW-LCC and CK groups at the class (**A**) and genus (**B**) levels. Wilcoxon rank-sum tests indicate significant differences at the family level (*, *p* < 0.05) (**C**). Species contribution of CAZyme families that were significantly enhanced (**D**).

The composition of CAZymes is analyzed at a class and family level in Figure 5A and B, with relative abundance of less than 1% categorized as other. There was a similar composition at the class and family levels of CAZymes between the BW-LCC and CK groups. It can be observed from Figure 5A that GTs, GHs, and CEs accounted for 94% of the relative abundance and were the major classes in both the BW-LCC and CK groups. GHs, GTs, and CEs can synergistically degrade cellulose and hemicellulose: GHs catalyze the breaking of glycosidic bonds to hydrolyze polysaccharides into oligosaccharides or monosaccharides, GTs facilitate glucose transfer between donor and acceptor molecules, and CEs are engaged in the degradation of polysaccharide side chains by removing ester bonds from the side chains [40]. GH146, CE11, GT30, GH37, PL22, and GT20 showed significant increases, with these changes being at the ** *p* < 0.05 level (Figure 5C). GH146, GH137, and PL22 have been shown to engage in the degradation of pectin in the human intestine [41]. Additionally, CE11, GT30, and GT20 participate in the synthesis of various glycans and lipids in microorganisms [42,43,44]. However, none of these families have been shown to be crucial for the degradation of lignocellulose in the human gut at present. Figure 5D shows the relationship between significantly elevated genes and gut microbes, of which the vast majority of these genes are derived from Bacteroidetes and Proteobacteria. Based on the sources of these families, it is apparent that the changing trend in families is related to the composition of the bacteria.

#### 3.4.2. The Role of BW-LCC in Causing the Enrichment of Lignin-Degrading Enzymes

**Figure 6:**
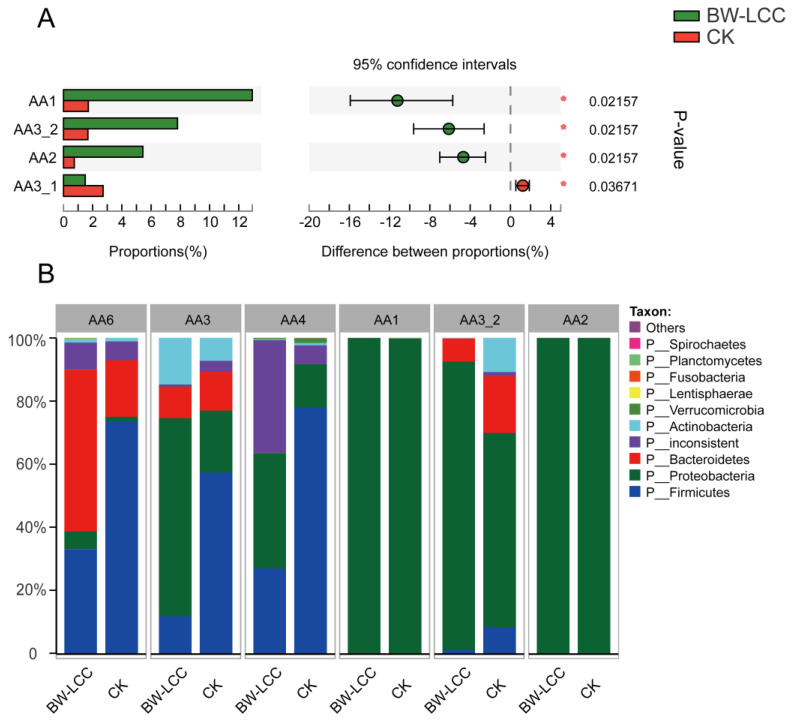
Relative abundance of AAs in gut microbes: (**A**) AAs with significant changes after in vitro fermentation, *, *p* < 0.05; (**B**) species contribution of AAs in gut microbes.

BW-LCC is rich in lignin and the degradation of lignin requires the involvement of AAs [20]. Abiotic degradation of lignocellulose is mediated by AAs via the Fenton reaction, which is capable of providing electrons and/or hydrogen peroxide to support other hydrolases during the degradation process [45]. Published reports have revealed that AAs are mainly concentrated in fungi. It was poorly documented that gut bacteria contained AAs, even though gut bacteria show considerable potential to degrade lignin [20]. With the aid of metagenomic analysis after fermentation, this study investigated changes in the dynamics of AAs in gut microbes. The results showed that there was a significant increase (* *p* < 0.05) in the relative abundance of AA1, AA2, and AA3_2 after fermentation with BW-LCC as substrate (Figure 6A). These results agree with previous reports that considered AA1, AA2, and AA3 to be the main enzymes involved in lignin degradation. Laccase (EC 1.10.3.2) is one kind of polyphenol oxidase which is common in AA1, and the copper ion in the active center enabling laccase to oxidize and dissolve polyphenols in lignin through electron exchange [46]. Oxidation of polyphenols is likely to produce phenolic aromatic aldehydes and acids that alter the pH in the environment [47]. Additionally, AA1 may collaborate with AA2 or other CAZymes to modify lignin degradation [48]. Peroxidases in AA2 are part of the superfamily of plant peroxidases that modify lignin [49]. AA2 has been reported to have three peroxidases related to lignin dissolution, which are manganese peroxidase (EC 1.11.1.13), lignin peroxidase (EC 1.11.1.14), and versatile peroxidase (EC 1.11.1.16) [50]. AA3_2 consisted of aryl alcohol oxidase (EC 1.1.3.7) and glucose 1-oxidase (EC 1.1.3.4), both of which may play a role in the modification of lignin by acting on the phenolic aromatic aldehydes and acids derived from lignin [20,51,52]. In addition, AA3_2 is likely to promote the synthesis of H_2_O_2_ in the intestine [50]. Records of AAs in the CAZy database are strongly biased toward the fungal kingdom, where catalysis reactions take place in an aerobic environment. Recent studies have demonstrated that some AAs can utilize H_2_O_2_ as an electron donor, and AAs have been discovered in anaerobic bacteria [53,54]. Intestinal flora might degrade lignin under anaerobic conditions through unique mechanisms based on the enrichment of three families in AAs. 

The most abundant AAs found in this study were AA6, AA4, AA3, AA1, AA2, and AA3_2. These families are widely distributed in intestinal bacteria, particularly Proteobacteria, Firmicutes, and Bacteroidetes. Bacteroidetes contributed some amount of AA6 (17.97%) and AA3 (12.23%), and Firmicutes contributed considerably to AA6 (73.54%), AA3 (57.35%), and AA4 (77.90%) in the CK group. In comparison, AA1, AA2, and AA3_2, which had a significant increase, were mainly derived from Proteobacteria. Due to the abundance of AA1, AA2, and AA3, Proteobacteria may have been able to exploit the complex lignin fraction in BW-LCC. Firmicutes also contained a certain number of AAs, but they were mostly AA6 and AA4, which did not differ after in vitro fermentation. The activities characterized by AA6 are all 1,4-benzoquinone reductases (EC. 1.6.5.6), which are involved in the metabolism of quinone, an intermediate metabolite of aromatic compounds that are commonly found in the plant kingdom especially in higher plants [55,56]. AA4 members are exclusively vanillyl-alcohol oxidases (EC 1.1.3.38), and it has been reported that they can oxidize vanillyl alcohol as a lignin unit to vanillin and further metabolize it [57]. It is likely that these enzymes require the conditions of AA1 and AA2 to function, which may explain their reduced abundance at the phylum level after fermentation. There has never been a report on the relationship between human intestinal Proteobacteria and AAs and lignin. In vitro fermentation with BW-LCC has demonstrated that human intestinal flora can utilize the lignin component of complex carbohydrates, in which Proteobacteria play an important role. 

## 4. Conclusions

This study examined the effects of BW-LCC on the microbiota of the human gut through in vitro fermentation. Overall, the characterization results of 2D-HSQC-NMR showed that BW-LCC contained GS-type lignin which accounting for over 70% as S units. BW-LCC fermentation generated a plentiful amount of acetic acid and propionic acid but was incapable of causing significant changes in other SCFAs, such as butyric acid and valeric acid. Additionally, 16s rRNA gene sequencing suggested that BW-LCC could greatly increase the relative abundance of Proteobacteria and decrease the F/B ratio. While this may result in intestinal inflammation, it may also prevent obesity. *Escherichia Shigella* and *Bacteroides* are the most variable genus after in vitro fermentation. These results indicate that BW-LCC can alter the composition of gut microbes. Additionally, BW-LCC promoted the enrichment of some CAZyme genes, especially AAs, most of which were derived from Proteobacteria. This indicates that the Proteobacteria in the human gut have the potential to degrade lignin. 

## Data Availability

The data used to support the findings of this study are available from the corresponding author upon request.

## Supplementary Materials

The following supporting information can be downloaded at: https://www.mdpi.com/article/10.3390/nu15010220/s1, Table S1: Signal attribution table for lignin and lignin–carbohydrate linkage bonds; Figure S1: The main linkage structures and structural units of the side chain and aromatic ring regions in the two-dimensional spectrum of this lignin sample; Figure S2: Rarefaction curves based on the Shannon index; Figure S3: Distribution of passing sequence versus subsequence.
