# Supplementary material for: In Vitro Fermentation of Beechwood Lignin–Carbohydrate Complexes Provides Evidence for Utilization by Gut Bacteria
Source: Nutrients. 2023 Jan 1;15(1):220. doi: 10.3390/nu15010220 (PMC9824187; doi:10.3390/nu15010220)
Supplement: Supplementary file 1 [file nutrients-15-00220-s001.zip › nutrients-2083940-supplementary.pdf]

Table S1. Signal attribution table for lignin and lignin-carbohydrate linkage bonds.

Lable	δC/δH(ppm)	Assignments
OCH3	56.4/3.70	C–H in methoxyl (OCH3)
Aγ	59.9/3.35-3.80	Cγ–Hγin β-O-4 substructures (A)
Aβ(S)	85.8/4.12	Cβ–Hβin β-O-4 linked to S(A)
Aα	71.8/4.86	Cα–Hαin β-O-4 unit (A)
Bγ	71.2/3.82-4.18	Cγ–Hγ in β-β resinol (B)
S2,6	103.9/6.70	C2,6–H2,6in syringyl units (S)
G2	110.8/6.97	C2–H2in guaiacyl units (G)
G5	114.5/6.70	C5–H5in guaiacyl units (G)
G6	119.0/6.78	C6–H6in guaiacyl units (G)
X1	101.5/4.25	C1–H1in β-D-xylopyranoside(X)
X2	72.5/3.05	C2–H2in β-D-xylopyranoside(X)
X3	73.91	C3–H3in β-D-xylopyranoside(X)
X4	75.3/3.56	C4–H4in β-D-xylopyranoside(X)
X5	63.2/3.24	C5–H5in β-D-xylopyranoside(X)
X5	63.2/3.94	C5–H5in β-D-xylopyranoside(X)

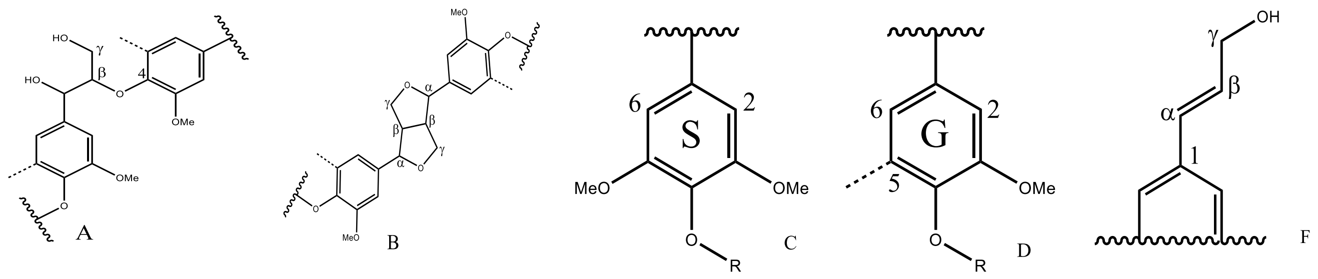

Figure S1. The main linkage structures and structural units of the side chain and aromatic ring regions in the two-dimensional spectrum of this lignin sample. (**A**), β-O-4 ether bond structure, the γ position is a hydroxyl group. (**B**), Resin alcohol structure, formed by linking β-β, α-O-γ and γ-O-α.(**C**), Syringyl structure. (**D**), Guaiacyl structure. (**E**), Phenylpropenol (end group). S, Syringyl. G, Guaiacyl.

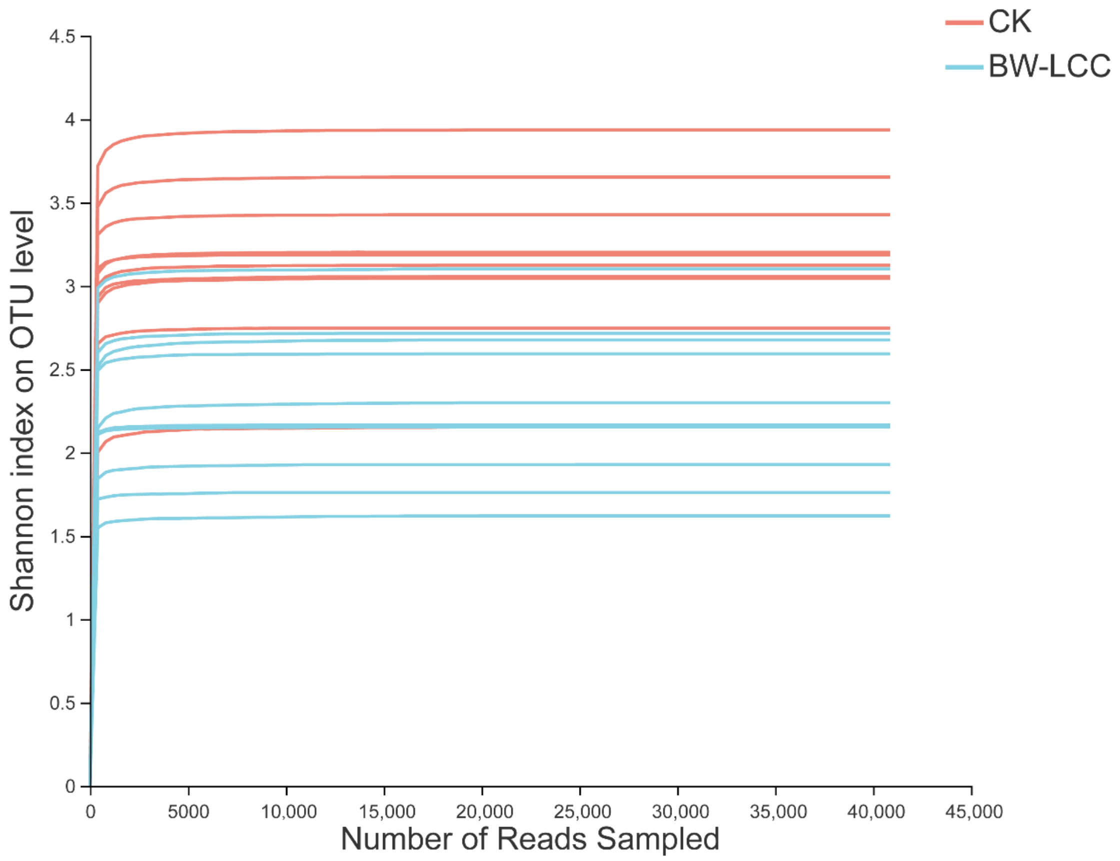

Figure S2. Rarefaction curves based on the Shannon index.

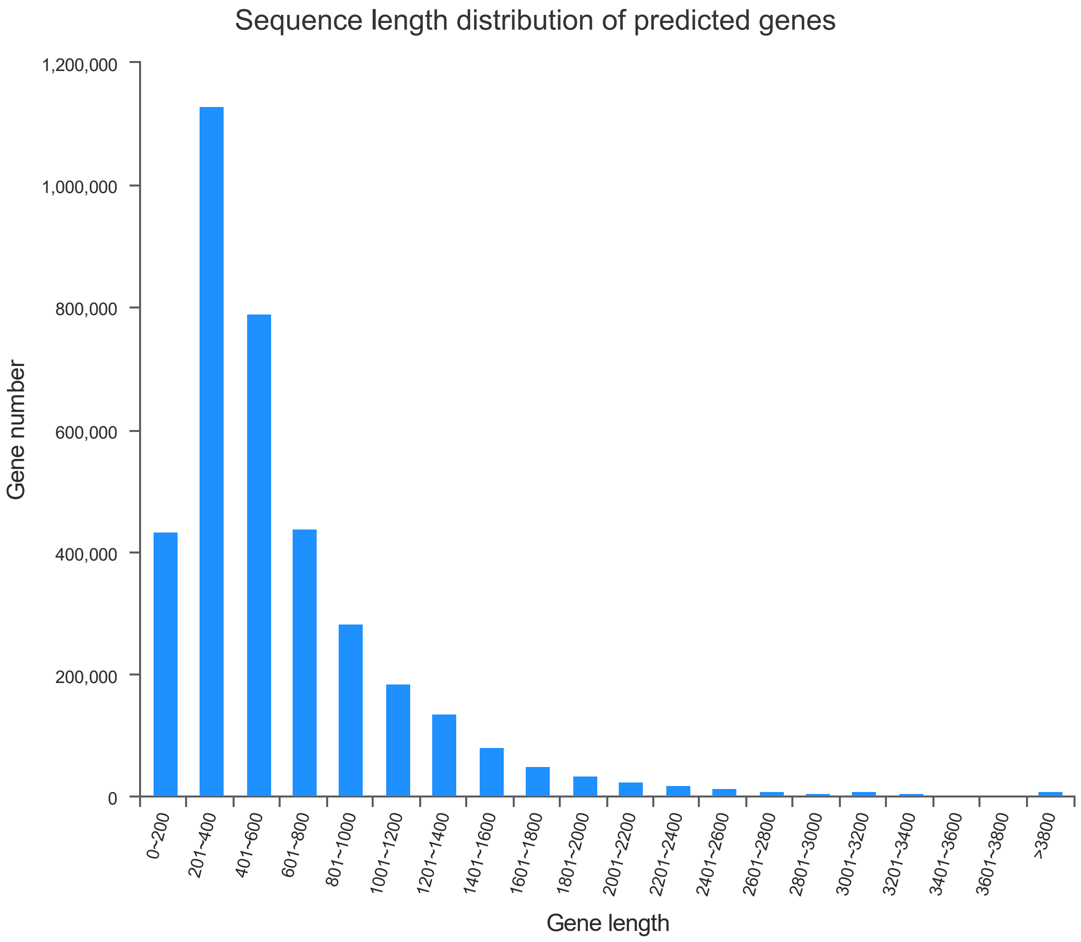

Figure S3. Distribution of passing sequence versus subsequence.
